# The burden of antimicrobial resistance in Iraq: an analysis from 2021

**DOI:** 10.1016/j.ijregi.2026.100976

**Published:** 2026-08-16

**Authors:** Celine Tabche, Zeenah Atwan, Hatham Atwan, Abbass A. Alhassani, Rafid A. Mohammed, Fakhria AlRashdi, Samiya Al-Khaldi, Ferdos AlaaAddin, Hamed Al Qanubi, Hazaa Al-Hinai, Ishani Sharma, Rishi Miriyala, Anees Nile, Faris Hassan Al-Lami, Ali R. Hashim, Ameen AlAlwany, David Rawaf, Ali Abutiheen, Jawad Ramadhan, Salman Rawaf

**Affiliations:** 1 School of Public Health, Imperial College London, London, UK; 2 Central Lab, College of Medicine, University of Basrah, Basrah, Iraq; 3 Department of Surgery, College of Medicine, University of Basrah, Basrah, Iraq; 4 Basrah Health Directorate, Basrah, Iraq; 5 Ministry of Health, Muscat, Sultanate of Oman; 6 Faculty of Pharmacy, Kunooz College, Basrah, Iraq; 7 WHO Collaborating Center for Public Health Education and Training, Imperial College London, London, UK.; 8 Faculty of Medicine, Imperial College London, London, UK; 9 Al-Nahreen Medical College, Baghdad, Iraq; 10 College of Medicine, University of Baghdad, Baghdad, Iraq; 11 Department of Internal Medicine, College of Medicine, University of Basrah, Basrah, Iraq; 12 College of Medicine, Kerbala University, Karbala, Iraq; 13 Department of Surgery, Al-Zahraa College of Medicine, University of Basrah, Basrah, Iraq

**Keywords:** Antimicrobial resistance, Infectious diseases, Disease burden, Antibiotic resistance, Iraq, Middle East

## Abstract

**Objectives:**

By 2050, antimicrobial resistance (AMR) is projected to become a leading cause of death globally, potentially surpassing cancer. Low- and middle-income countries are particularly vulnerable due to fragile health systems, instability, and economic constraints.

This study assessed the burden of AMR in Iraq, identifying the principal infectious syndromes and pathogens contributing to mortality and disability-adjusted life years (DALYs) using Global Burden of Disease data.

**Methods:**

Data on AMR in Iraq were extracted from the Global Burden of Disease (GBD) study and compared with data for the North Africa and Middle East (NAME) region.

**Results:**

In 2021, the leading infectious causes of death in Iraq were bloodstream infections (BSIs), lower respiratory infections (LRIs), peritoneal and abdominal infections, tuberculosis, and urinary tract infections (UTIs), including pyelonephritis. AMR contributed 63.9%, 55%, 58%, 6.9%, and 62.5% of these deaths, respectively. The syndromes responsible for DALYs were BSIs, LRIs, upper respiratory tract infections, diarrhoeal diseases, and peritoneal and abdominal infections; AMR accounted for 60%, 55%, 0%, 12.5%, and 58.9% of DALYs, respectively.

The principal pathogens associated with deaths were *Streptococcus pneumoniae* (12.9%), *Staphylococcus aureus* (12.4%)*, Klebsiella pneumoniae* (9.9%)*, Acinetobacter baumannii* (8.1%)*, and Pseudomonas aeruginosa* (7.2%). Mortality rates exceeded those of the North Africa and Middle East (NAME) region for most syndromes, notably UTIs and pyelonephritis.

**Conclusion:**

AMR represents a substantial burden on mortality and disability in Iraq, with the highest overall burdens reported for BSIs and LRIs. Coordinated national interventions are required in Iraq, including strengthened diagnostics, workforce training, public awareness, and regulatory enforcement.

## Highlights

• First study to quantify the antimicrobial resistance (AMR) burden in Iraq using 2021 Global Burden of Disease data.
• Bloodstream infections are the top cause of infectious death in Iraq.
• AMR accounts for 63.9% of deaths attributed to bloodstream infections.
• Urinary tract infections (UTI) mortality in Iraq is tenfold higher than the regional average.
• *Streptococcus pneumoniae* and *Staphylococcus aureus* are the primary pathogens causing death.

## Introduction

Antimicrobial resistance (AMR) is a critical global health threat. Based on the Global Burden of Disease (GBD) study, nearly 4.71 million deaths were associated with bacterial AMR, including about 1.14 million attributed to it, with apparent disparities in trends between children and adults [1]. By 2050, AMR could cause an estimated 10 million deaths annually, surpassing cancer [2]. Most available antibiotics were discovered between the 1940s and 1970s, and consequently, AMR appeared [3]. Following the 1980s, there was a significant decline in the discovery of new antibiotics, which failed to keep pace with the rising rate of antibiotic resistance [3]. Misuse and inappropriate prescription of antibiotics, use of antibiotics against viral infections, incomplete course use, self-medication, and use of antibiotics in agriculture, animal farming, and fisheries are poorly regulated. These factors, along with inadequate waste disposal and poor sanitation, contribute to the spread of multidrug-resistant (MDR) bacteria, particularly gram-negative bacteria (GNB) [4].

More than 95% of bacterial genomes harbor resistance genes against glycopeptides, macrolides, tetracyclines, and disinfectants [5]. These genes, found on both chromosomes and plasmids, contribute to the spread of resistance [6]. The Bacterial Priority Pathogens List (BPPL) announced by the WHO comprises 24 pathogens and covers 15 families of antibiotic-resistant bacterial pathogens. GNB are at the top of the list [7].

AMR is particularly concerning in low- and middle-income countries due to its significant impact on health and the economy. The situation is exacerbated by the widespread misuse of antibiotics, particularly in the Middle East and EMR region, where self-medication rates exceed 80% and reach 98%. Common sources of antibiotics include pharmacies that provide them without prescriptions, leading to inappropriate use [8]. Penicillin is the most used antibiotic in the region, while adherence to antibiotic policy guidelines among physicians remains low [9].

In Iraq, the situation is worsened by the over-the-counter availability of antibiotics, compounded by years of conflict, economic sanctions, and inadequate training for healthcare providers. This has led to widespread resistance, particularly among bacteria such as *Escherichia coli, Klebsiella pneumoniae, Pseudomonas aeruginosa, Acinetobacter baumannii, Salmonella spp., Streptococcus pneumoniae*, and *Staphylococcus aureus*. Resistance to third-generation cephalosporins and, to a lesser extent, carbapenems has been identified, with increasing resistance in *P. aeruginosa* and *A. baumannii*. Methicillin-resistant *S. aureus* (MRSA) is reported to affect over 60% of patients in Iraq [10].

This study analyses the disease burden associated with and attributable to AMR in Iraq, focusing on deaths and Disability-adjusted life years (DALYs) in 2021, and compares these results with those for the broader NAME region from the GBD study.

## Methods

Data on AMR in Iraq were extracted from the GBD study [11], a comprehensive assessment of disease, injury, and risk factors worldwide that provides the most recent epidemiological estimates for 371 diseases and injuries across 21 GBD regions and 204 countries and territories from 1990 to 2021. The GBD methodology provides estimates for deaths, years of life lost (YLLs), and years lived with disability (YLDs) for a variety of health conditions. DALYs are a standard metric for assessing disease burden, representing the total healthy years lost from disease onset to death. This measure combines years of life lost (YLLs) due to premature mortality and YLDs. The GBD study also addresses the positive or negative correlations between independent variables (covariates) and diseases and conditions. These covariates comprise socioeconomic factors, demographic variables, climate, food intake, and health system access [11].

The GBD study categorizes diseases and injuries into four main levels. AMR is a complex contributor to disease burden because it influences the severity and outcomes of many infections. AMR is defined by the development of the capacity of bacteria, fungi, parasites, or viruses to stop or mitigate the action of previously efficacious antibiotics, antifungal, antiviral, and other medications. Such resistance worsens outcomes and increases morbidity and mortality [4].

The breakdown of the GBD hierarchy and where the impact of AMR fits can be described as follows:

The GBD hierarchy starts with the broadest categories at Level 1 and becomes increasingly specific:

• **Level 1: Three Major Cause Groups**: Communicable, Maternal, Neonatal, and Nutritional Diseases (Group I), Non-Communicable Diseases (Group II), Injuries (Group III)
• **Level 2: Major Disease/Injury Subgroups** (Infectious diseases, Cancers, Cardiovascular diseases)
• **Level 3: Disease/Injury Categories** (Enteric infections, Ischemic heart disease, Road injuries)
• **Level 4: Specific, Detailed Causes of Health Loss** (Typhoid fever, Ischaemic stroke, Pedestrian Road injury) [11]

Based on how the GBD framework handles similar concepts, AMR is generally estimated and categorized in relation to **Level 4** causes, often associated with a **Level 3** category.

In terms of Cause of Death/Disability, AMR's burden is measured by calculating the deaths and DALYs that are **attributable to** drug-resistant infections. These resistant infections would fall under **the Level 4 category, which is** nested within the **Level 3** category of Infectious diseases, which in turn is under the **Level 1** category of *Communicable, Maternal, Neonatal, and Nutritional Diseases* [12].

The Global Research on Antimicrobial Resistance Project was established to quantify the burden. In these comprehensive analyses, AMR is often treated as a factor *contributing* to death from an underlying infection, like how other **Risk Factors** (such as smoking or high blood pressure) are modeled in the GBD. This involves calculating the **Population Attributable Fraction** to determine the proportion of the disease burden attributable to resistance [13].

GBD estimates the burden of AMR associated with/attributable to 11 of the 22 infectious syndromes included in the framework: bloodstream infections, meningitis, lower respiratory infections, endocarditis, peritoneal and intra-abdominal infections, diarrhoeal diseases, urinary tract infections and pyelonephritis, bone and joint infections, skin and subcutaneous tissue infections, tuberculosis, and typhoid/paratyphoid fever, together with invasive non-typhoidal Salmonella infections. GBD analysis covers 22 types of bacterial pathogens and 16 antibiotic drug classes or combinations for which resistance was evaluated [1].

The analysis in this study focuses on major deaths and DALYs attributed to infectious syndromes, including BSIs, LRIs, and peritoneal and abdominal infections. The study also calculated the percentages of AMR-related deaths and DALYs in each syndrome in Iraq, ranked the most common antibiotic-resistant pathogens, and calculated the percentages of representation of the top five pathogens in Iraq. Finally, trends in deaths and DALYs were compared with those of the NAME region.

GBD data are obtained from primary and secondary sources. Primary data are collected directly through household surveys, health facility assessments, medical record reviews, verbal autopsies, biometric measurements, and other field-based studies conducted in collaboration with local partners. These data are gathered using standardized protocols and are subsequently made available through the Global Health Data Exchange (GHDx).

Most GBD estimates, however, rely on secondary data obtained from existing sources such as censuses, vital registration systems, disease registries, demographic surveillance systems, hospital records, administrative databases, scientific literature, and national surveys. The Institute for Health Metrics and Evaluation (IHME) systematically identifies, acquires, catalogs, and extracts these datasets through the GHDx, then integrates them into GBD models to generate comparable health estimates across countries and time periods [[14], [15], [16], [17], [18], [19]].

## Results

### Deaths attributable to AMR by infectious syndromes in Iraq

In 2021, GBD data revealed that the top five causes of death from infectious syndromes in Iraq were BSI, LRIs, peritoneal and abdominal, tuberculosis, and UTI and pyelonephritis, with counts of 10,202.26, 729.91, 1,253.12, 900.45, and 878.47, respectively. AMR accounted for 63.9%, 55%, 58%, 6.9%, and 62.5% of deaths attributed to BSI, LRIs, peritoneal and abdominal, tuberculosis, UTI and Pyelonephritis, respectively. Other infections contributing to mortality include skin infections, diarrhea, encephalitis, meningitis, hepatitis, endocarditis, Myo-/Peri/Carditis, sexually transmitted infections (STIs), typhoid and paratyphoid, bones, joints and related infections, myelitis, meningoencephalitis, and other central nervous system infections (MMO), upper respiratory, genital, parasitic, oral and eye infections (Figure 1A and B).

**Figure 1:**
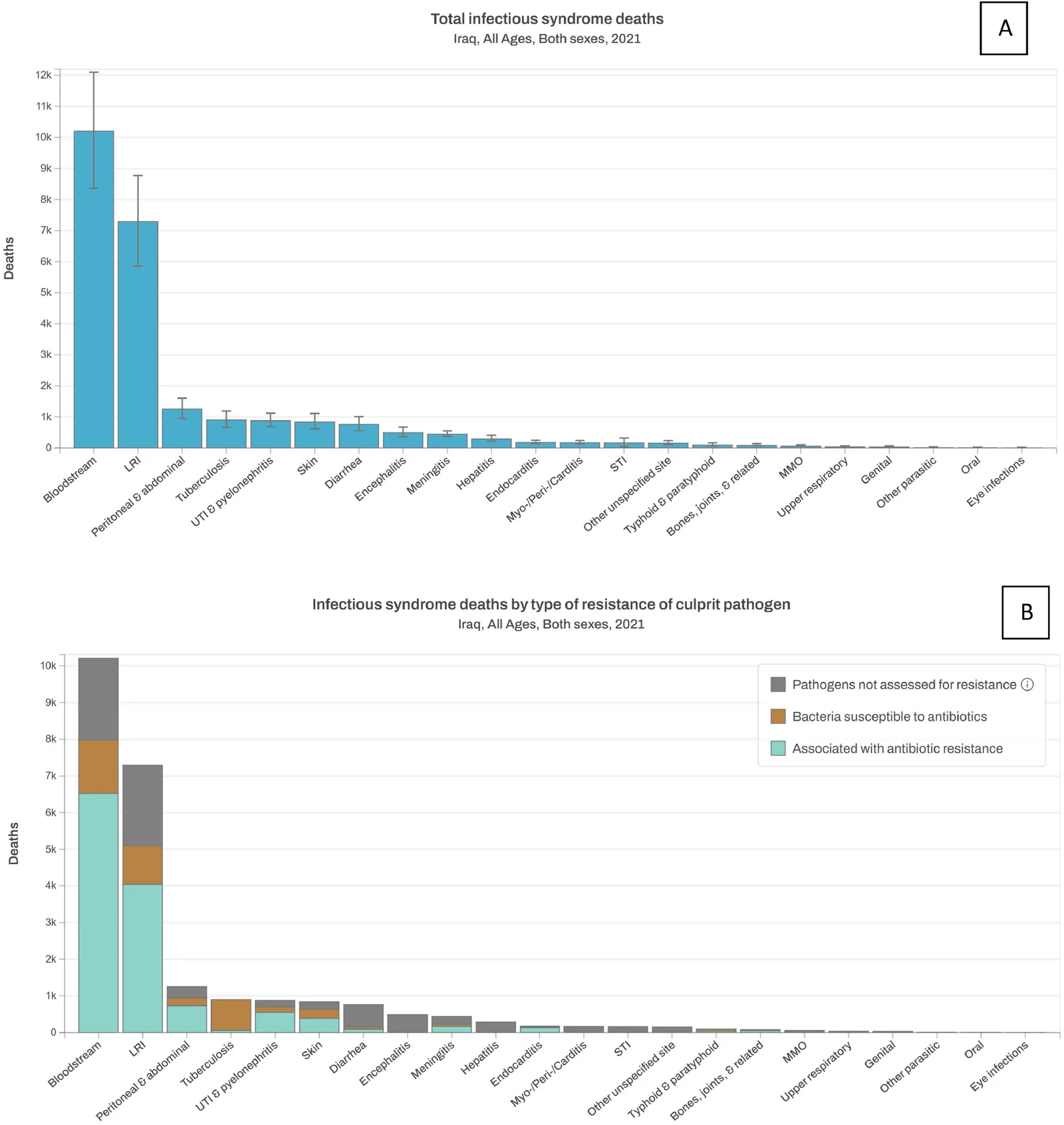
(A and B); A: Deaths of infectious syndromes attributed to untested, susceptible, and resistant microorganisms in Iraq, 2021. Source of data: [11]. B: Deaths attributed to AMR in different infectious syndromes in Iraq, 2021. Source of data:[11].

### DALYs attributable to AMR by infectious syndromes in Iraq

GBD data reported that the top five infectious syndromes causing DALYs in Iraq were BSI, LRIs, upper respiratory, diarrhea, and peritoneal and abdominal, with the highest at 480052.98, 280759.44, 119956.16, and 75306.81, and the lowest at 42405.28, respectively. AMR accounted for 60%, 55%, 0%, 12.5%, and 58.9% of DALYs attributed to BSI, LRIs, upper respiratory, diarrhea, and peritoneal and abdominal infections, respectively. Other syndromes contributing to DALYs included encephalitis, tuberculosis, meningitis, skin, UTI, and pyelonephritis, bones and joints and related diseases, STI, hepatitis, unspecified sites, myo/peri/carditis, typhoid and paratyphoid, endocarditis, MMO, parasitic infections, oral, genital, and eye infections (Figure 2A and B).

**Figure 2:**
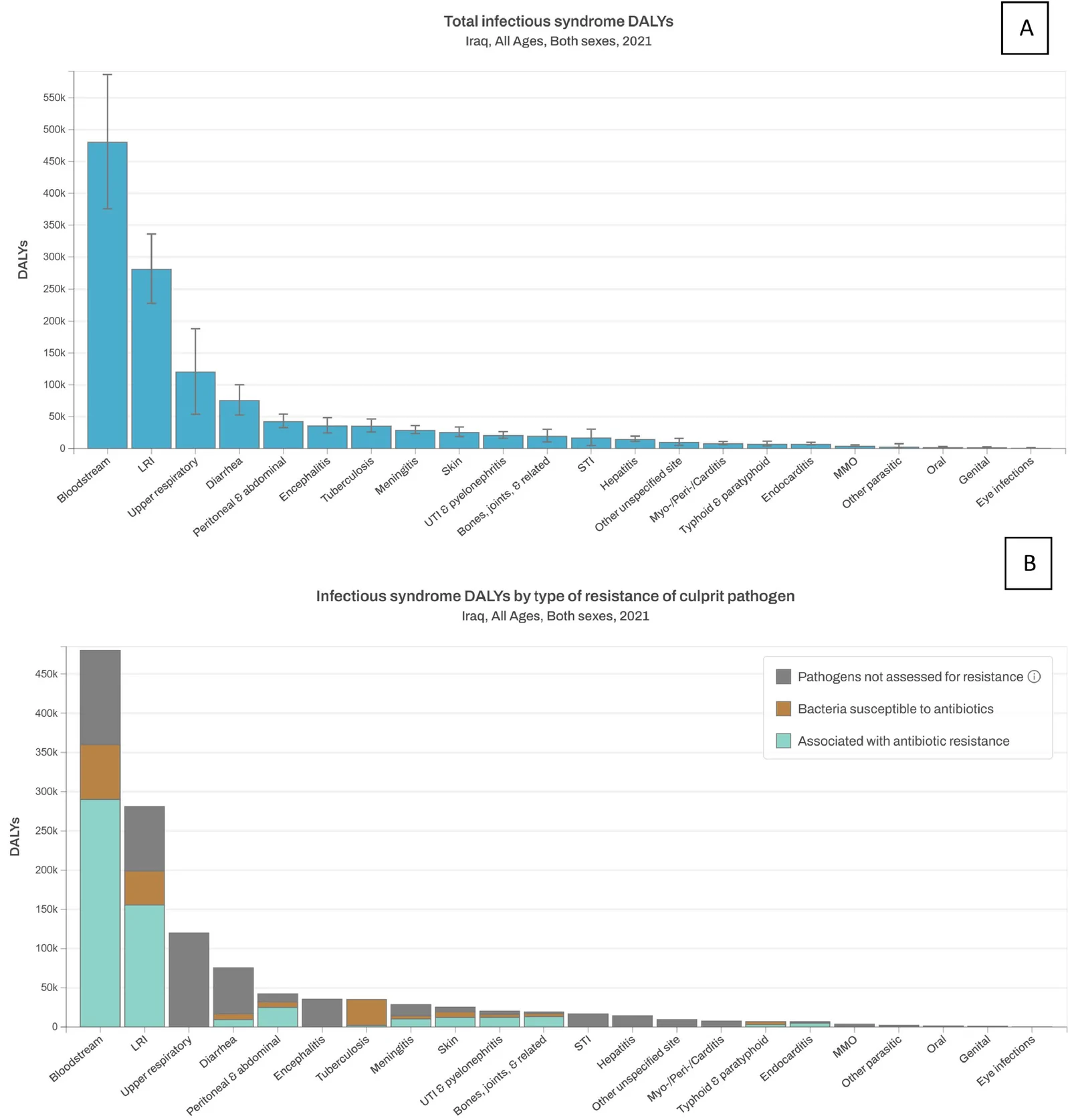
(A and B); A: DALYs of infectious syndromes attributed to untested, susceptible, and resistant microorganisms in Iraq, 2021. Source of data: [11]. B: DALYs attributed to AMR in different infectious syndromes in Iraq, 2021. Source of data: [11].

### Deaths and DALYs attributable to specific pathogens

The top five bacterial species were responsible for deaths as the top five pathogens, with *S. pneumoniae* (12.9%) and *S. aureus* (12.4%), followed by *A. baumannii*, P*. aeruginosa*, and *K. pneumoniae* at percentages of 8.1%, 7.2%, and 9.9 %, respectively (Figure 3A and B). A similar trend was observed for the leading pathogens responsible for DALYs. *S. pneumoniae* (19%), *S. aureus* (1.5%), and *A. baumannii* (1%) were the top three pathogens, similar to those that caused deaths in Iraq in 2021. *K. pneumoniae* ranked fourth preceding *P. aeruginosa*, but both shared comparable percentages, 0.96 and 0.94%, respectively, Figure 3 (A and B).

**Figure 3:**
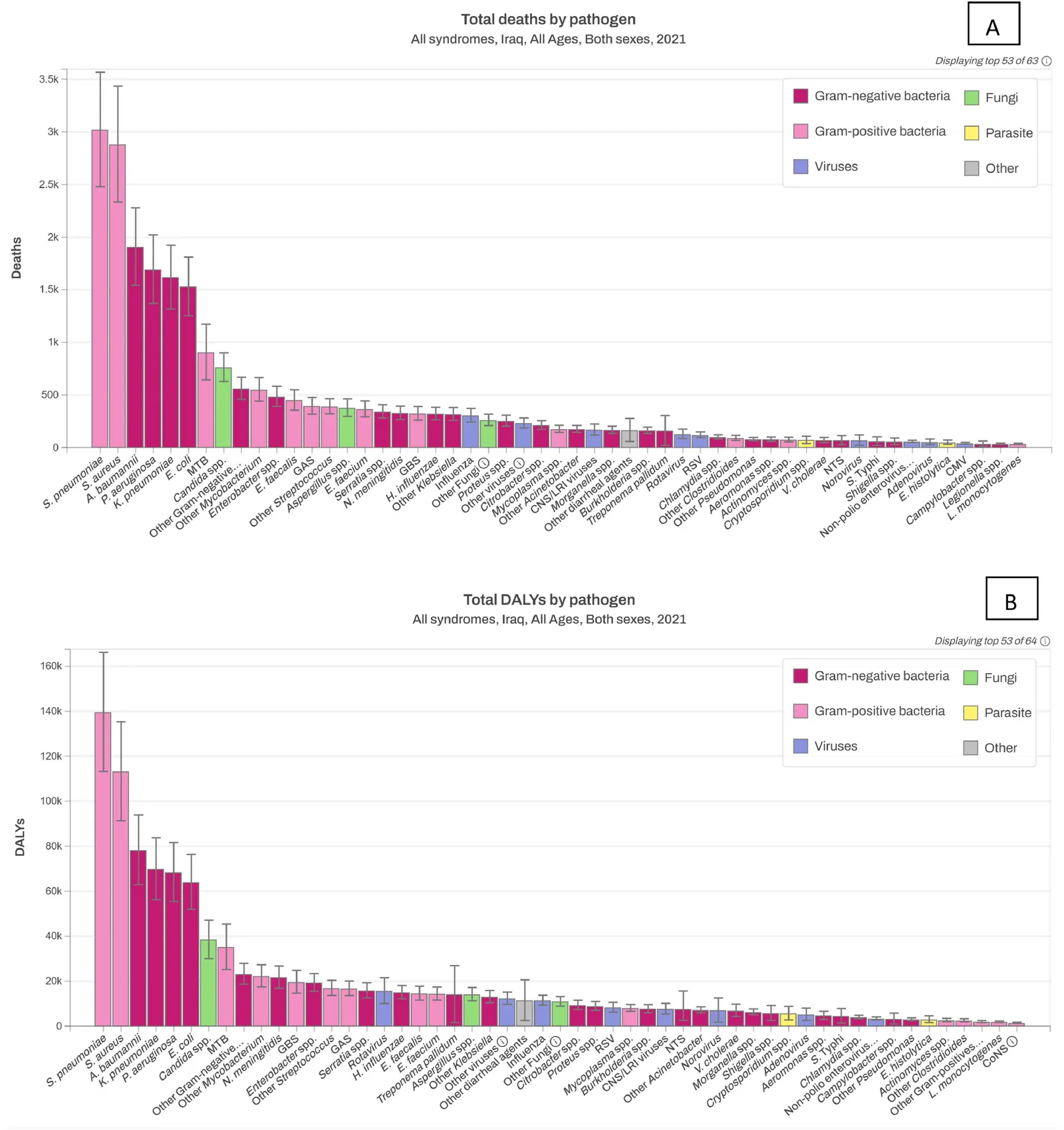
(A and B); A: Total deaths associated and attributable to specific pathogens in Iraq, 2021. Source of data: [11]. B: Total DALYs attributable to specific pathogens in Iraq, 2021. Source of data: [11].

### Iraq deaths and DALYs data compared with the NAME region

Consistent trends were observed in the top five infectious syndromes in the NAME region, compared with those in Iraq. Concerning AMR data, slightly higher deaths due to AMR at 63.9%, 55%, and 58% were reported in Iraq compared to 55%, 48%, and 55.6% in NAME for the BSI, LRI, and peritoneal and abdominal syndromes. Re-deaths attributed to AMR in tuberculosis between Iraq and NAME were comparable. However, about tenfold higher percentages in UTI and pyelonephritis deaths were noticed in Iraq compared to the NAME region (Figure 4). DALYs data were slightly comparable between Iraq and NAME in tuberculosis, UTI, and pyelonephritis. No AMR data were reported for upper respiratory tract infections in either Iraq or NAME. Finally, a slightly higher percentage of AMR in BSI and LRI DALYs in Iraq compared to the NAME region (Figure 5).

**Figure 4:**
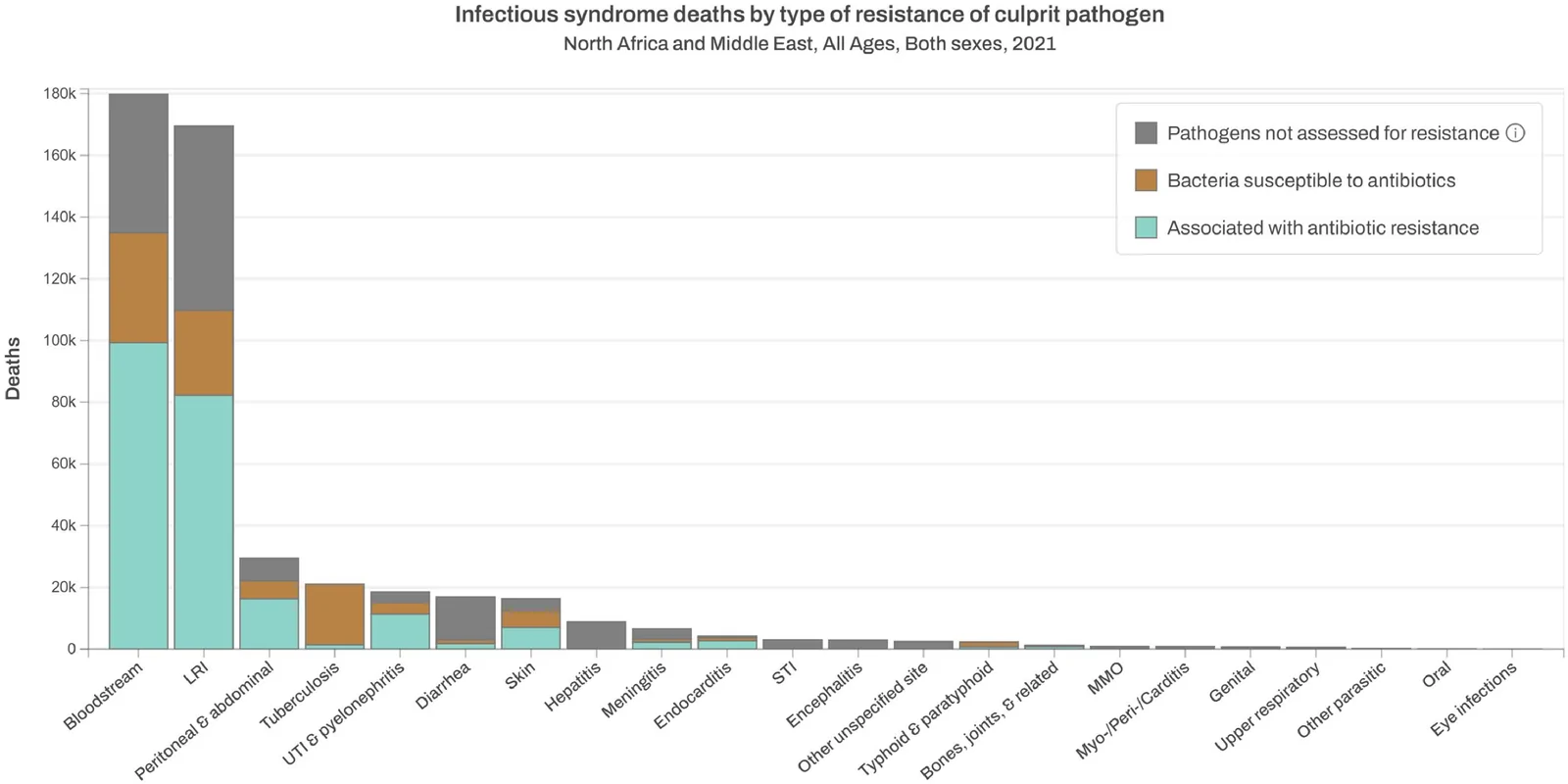
Total deaths associated and attributable to specific pathogens in NAME, 2021. Source of data: [11].

**Figure 5:**
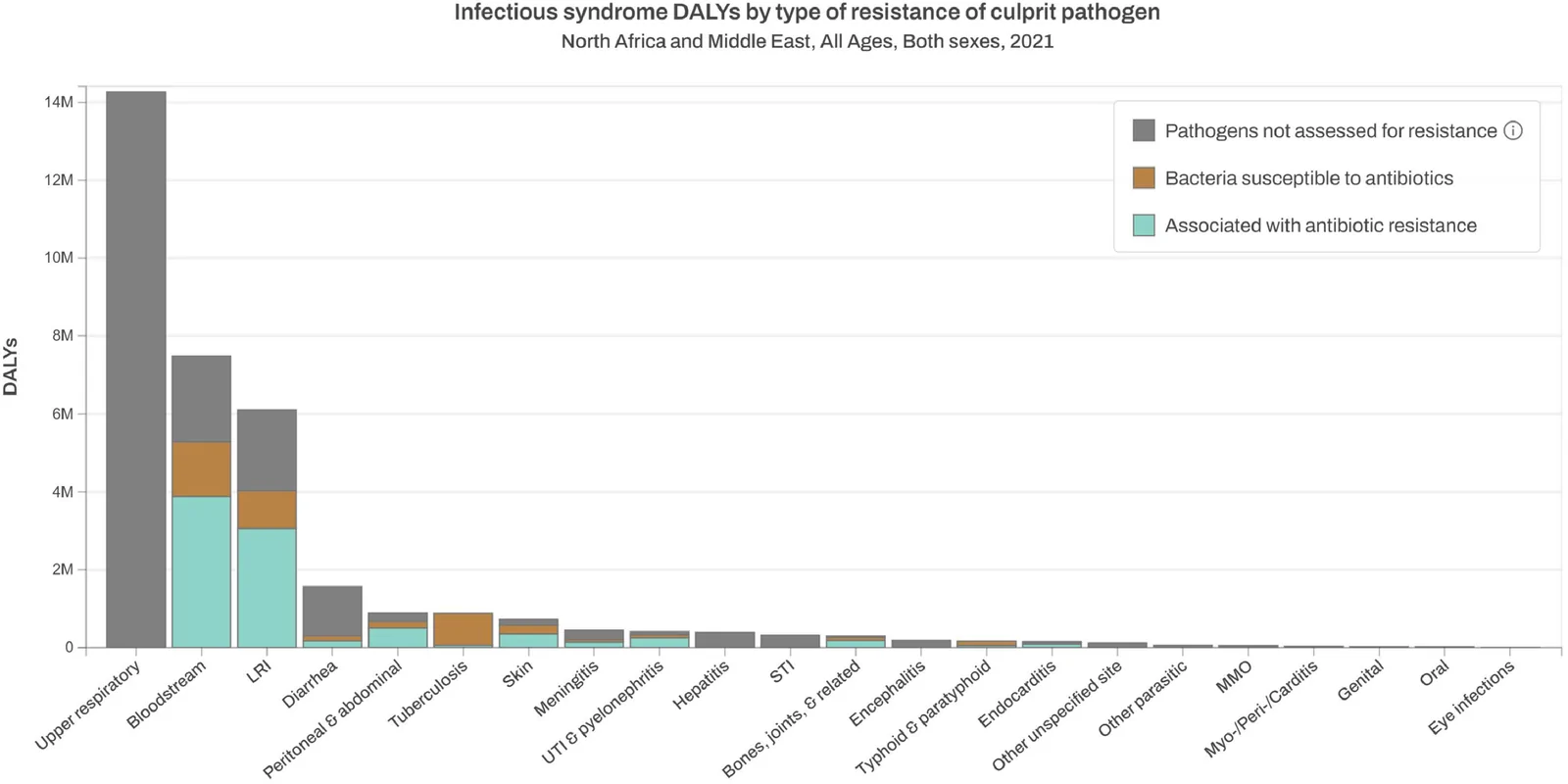
Total DALYs associated and attributable to specific pathogens in NAME, 2021. Source of data: [11].

## Discussion

To our knowledge, this study is the first to quantify the burden of AMR in Iraq and compare it with that of the broader NAME region. Death and DALYs results showed the top five reasons for deaths due to infectious syndromes in Iraq were BSI, LRIs, peritoneal and abdominal infections, tuberculosis, and UTI and pyelonephritis, with AMR accounting for 63.9%, 55%, 58%, 6.9%, and 62.5%, respectively. The top five causes of DALYs in Iraq were BSI, LRIs, upper respiratory infections, diarrhea, and peritoneal and abdominal infections, with AMR accounting for 60%, 55%, 0%, 12.5%, and 58.9%, respectively. Despite higher percentages of the top deaths and DALYs causes, these findings align with studies conducted across Asia, the Middle East, and Iraq [20]. BSI has a significant impact on mortality, with gram-negative, extensively drug-resistant (XDR) bacteria posing strong independent risk factors for death [21]. BSI is diagnosed when a patient has positive blood cultures and systemic signs of infection. It can either be secondary to a known source or primary with no identified origin. BSIs account for 40% of community-acquired and hospital-acquired sepsis and septic shock cases, and approximately 20% of those acquired in the ICU [22]. Approximately 15% of deaths were attributed to BSI, with 21.9% of cases involving carbapenem-resistant GNB [20]. BSIs caused by multidrug-resistant (MDR) GNB are associated with poor clinical outcomes and a substantial impact on quality of life [23].

In Iraq, MDR bacteria, including Gram-positive (GP) and Gram-negative (GN) strains resistant to ampicillin, erythromycin, levofloxacin, tetracycline, and trimethoprim, are commonly isolated from patients with LRTI [24]. LRI is often self-treated, with the public frequently obtaining antibiotics based on advice from family, friends, or pharmacists. Knowledge about the viral origins of many LRI cases is limited within the Iraqi community, even among some healthcare providers, which leads to inappropriate antibiotic use. It has been shown that poor knowledge among Iraqi people regarding proper antibiotic use is common. Additionally, poor attitudes of pharmacists and physicians regarding antibiotic prescribing were reported [25]. The prominence of peritoneal and abdominal infections among the top syndromes is consistent with global data indicating a 30% rate of antibiotic-resistant bacteria at the time of initial surgery [26].

Local factors such as wars and conflicts, displaced populations, the increased number of private hospitals, and the rise in surgical procedures, particularly cosmetic surgeries in private clinics, as well as severe climate change [27], have significantly contributed to the rise of MDR microbes. Lifestyle in Iraq after 2003 has totally changed due to socioeconomic status; obesity increased significantly, and this could alter the microbiome community in obese individuals, resulting in increased AMR impact [28]. An additional factor may be religious festivals, which could facilitate the exchange of resistant bacterial strains, particularly respiratory bacteria. The top five bacterial species accounted for deaths as the top five pathogens: *S. pneumoniae* (12.9%) and *S. aureus* (12.4%), followed by *A. baumannii, P. aeruginosa*, and *K. pneumoniae*, at percentages of 8.1%, 7.2%, and 9.9%, respectively. A consistent pattern was observed among the top pathogens responsible for DALYs: *S. pneumoniae* (19%), *S. aureus* (1.5%), and *A. baumannii* (1%) were the top three pathogens, similar to those that caused deaths in Iraq in 2021. *K. pneumoniae* ranked fourth preceding *P. aeruginosa*, but both shared comparable percentages, 0.96 and 0.94%, respectively.

This aligns with data from Najaf, Iraq, where *Pseudomonas aeruginosa*, *S. aureus*, *K. pneumoniae*, *Staphylococcus epidermidis*, *A. baumannii*, and *Enterobacter cloacae* were frequently found, including methicillin-resistant *S. aureus* and extended-spectrum beta-lactamase-producing Enterobacteriaceae [29]. Furthermore, MDR bacterial strains were identified in Mosul in patients admitted with injuries from violent conflicts between 2018 and 2019, with MRSA and extended-spectrum beta-lactamase Enterobacteriaceae being prevalent at rates of 53% and 33.2%, respectively [30].

Across the NAME region, resistance patterns are similar, with S. aureus among the predominant resistant Gram-positive pathogens, while E. coli and K. pneumoniae show high levels of Gram-negative resistance. This could be due to the fact that similar factors play a role in AMR development in the region. Self-medication with antibiotics, especially for the upper respiratory tract, is common in this region due to socio-cultural, economic, and regulatory factors that drive this [31]. As documented in Lebanon, GNBs exhibit resistance to third-generation cephalosporins and carbapenems [32,33]. MDR GP and GN bacteria are acquired in both community and healthcare settings, with a higher likelihood of resistant strains, such as E. coli, emerging in nosocomial infections. High mortality rates are often associated with ICU-acquired MDR GN BSI infections [34]. Resistance in *S. aureus*, enterococci, and GN bacilli correlates with higher mortality, increased morbidity, extended hospital stays, higher healthcare costs, and poorer outcomes [35]. GNBs’ resistance to carbapenems spreads rapidly via horizontal transfer of carbapenemase-encoding genes, thereby limiting treatment options and increasing the risk of outbreaks [36]. Additionally, carbapenem-resistant A. baumannii infections show higher mortality rates than those with carbapenem-susceptible strains [37].

The emergence of MDR and XDR bacterial isolates in Iraq has also been influenced by environmental factors, including pollution and climate change, and has been exacerbated by the COVID-19 pandemic. Resistance in bacteria can develop naturally or be acquired through changes in the bacterial genetic code in response to environmental pressures, with approximately 20% of chromosomal or plasmid DNA involved [38]. Pollution with heavy metals in Iraq may serve as a co-selection factor in the development of antibiotic resistance in various GNB species, such as *E. coli* and *P. aeruginosa* [39]. Antibiotic misuse in Iraq is widespread, with misuse rates reaching 92% due to insufficient public knowledge about antibiotic misuse risks and non-evidence-based prescribing practices by some physicians and pharmacists, which reaches 98% of pharmacists who sell antibiotics without prescription [8,25,40]. Antibiotics are regularly prescribed empirically by physicians in Iraqi public hospitals, whereas sales of antibiotics by pharmacists are uncontrolled [40]. Evidence showed the presence of antibiotics in raw meat and drinking water [40]. The use of antibiotics in livestock is another major factor in the development of antibiotic resistance among the public, necessitating the adoption of the “One Health” concept [41]. Uncontrolled use of antibiotics in animals selects for and drives the evolution of antibiotic-resistant genes that can be transmitted to humans via zoonoses, including well-known resistant species such as *K. pneumoniae, A. baumannii*, and other bacteria implicated in nosocomial infections. Fish may also contribute to AMR in the Iraqi local environment, as growing evidence indicates the presence of resistant strains isolated from fish breeding farms [42].

Policymakers need to introduce immediate public health and regulatory measures to reduce the misuse of antibiotics. However, the Iraqi Ministry of Health initiated a national action plan to address antimicrobial resistance. In addition, a national coordinating center (NCC) for antimicrobial resistance within the public health directorate was established in 2017 to strengthen efforts to combat AMR. These include training physicians in effective prescribing, prohibiting dispensing without a prescription from licensed physicians, restricting the use of antibiotics in agriculture, animal farming, and fisheries, recommending microbial genome sequencing as a practical approach for controlling AMR, mapping areas with widespread AMR, developing a tracking system for antibiotic prescriptions and increasing public awareness of the danger of misuse of antibiotics. In addition, enforcement of restrictions on non-prescription antimicrobial dispensing is needed, as studies from Iraq report that more than 50% of antimicrobials may be obtained without a prescription [43].

This study had certain limitations due to the reliance on Global Burden of Disease data from 2021 only, with antibiotic misuse in Iraq predating this by several decades, especially after the Gulf War. Hospitalized patients, particularly those with burn injuries, are especially vulnerable to nosocomial infections. Many studies, master’s theses, and PhD dissertations have investigated bacterial resistance, but most remain unpublished or are published in predatory journals, limiting access to their findings. This study focused only on specific infectious syndromes and bacterial species, potentially overlooking other significant contributors to AMR in Iraq. Future research should emphasize the social and cultural factors that influence antibiotic misuse.

## Conclusion

The results showed that AMR is a driver of mortality in Iraq, particularly BSI, respiratory, and abdominal infections. *S. pneumoniae* and *S. aureus* were the leading pathogens. AMR poses a significant public health challenge in Iraq, exacerbated by issues such as the national health system and various social and cultural contributors, with estimates that Iraq surpasses other countries in AMR estimates.

Iraq urgently needs coordinated health and economic interventions, including public awareness campaigns, healthcare provider training, and strengthened legal regulations to address this escalating challenge. Patient education on proper antibiotic use is essential, as it underscores the importance of timely administration, appropriate dosing, and antibiotic sensitivity testing to identify the most effective treatment options and potential alternatives. Further studies concerning AMR in Iraq are urgently needed. Finally, reducing the overuse of disinfectants in healthcare settings is crucial, as it may contribute to the emergence of resistant strains. This study would benefit from including data on the range of antibiotics to which bacterial species exhibit resistance, as well as more detailed data on MDR species.

## CRediT authorship contribution statement

**Celine Tabche:** Conceptualization, Data curation, Formal analysis, Investigation, Methodology, Project administration, Supervision, Validation, Visualization, Writing – original draft, Writing – review & editing. **Zeenah Atwan:** Conceptualization, Data curation, Formal analysis, Investigation, Methodology, Supervision, Validation, Visualization, Writing – original draft, Writing – review & editing. **Hatham Atwan:** Formal analysis, Validation, Writing – review & editing. **Abbass A. Alhassani:** Validation, Writing – review & editing. **Rafid A. Mohammed:** Validation, Writing – review & editing. **Fakhria AlRashdi:** Validation, Writing – review & editing. **Samiya Al-Khaldi:** Validation, Writing – review & editing. **Ferdos AlaaAddin:** Validation, Writing – review & editing. **Hamed Al Qanubi:** Validation, Writing – review & editing. **Hazaa Al-Hinai:** Validation, Writing – review & editing. **Ishani Sharma:** Validation, Writing – review & editing. **Rishi Miriyala:** Validation, Writing – review & editing. **Anees Nile:** Validation, Writing – review & editing. **Faris Hassan Al-Lami:** Validation, Writing – review & editing. **Ali R. Hashim:** Validation, Writing – review & editing. **Ameen AlAlwany:** Validation, Writing – review & editing. **David Rawaf:** Validation, Writing – review & editing. **Ali Abutiheen:** Validation, Writing – review & editing. **Jawad Ramadhan:** Validation, Writing – review & editing. **Salman Rawaf:** Conceptualization, Supervision, Writing – review & editing.

## Declaration of competing interest

The authors have no competing interests to declare.
